# Different patterns of white matter microstructural alterations between psychotic and non-psychotic bipolar disorder

**DOI:** 10.1371/journal.pone.0265671

**Published:** 2022-03-18

**Authors:** Dong-Kyun Lee, Hyeongrae Lee, Vin Ryu, Sung-Wan Kim, Seunghyong Ryu

**Affiliations:** 1 Department of Mental Health Research, National Center for Mental Health, Seoul, Republic of Korea; 2 Department of Psychiatry, Chonnam National University Medical School, Gwangju, Republic of Korea; Beijing Institute of Basic Medical Sciences, CHINA

## Abstract

This study aimed to investigate alterations in white matter (WM) microstructure in patients with psychotic and non-psychotic bipolar disorder (PBD and NPBD, respectively). We used 3T-magnetic resonance imaging to examine 29 PBD, 23 NPBD, and 65 healthy control (HC) subjects. Using tract-based spatial statistics for diffusion tensor imaging data, we compared fractional anisotropy (FA) and mean diffusion (MD) pairwise among the PBD, NPBD, and HC groups. We found several WM areas of decreased FA or increased MD in the PBD and NPBD groups compared to HC. PBD showed widespread FA decreases in the corpus callosum as well as the bilateral internal capsule and fornix. However, NPBD showed local FA decreases in a part of the corpus callosum body as well as in limited regions within the left cerebral hemisphere, including the anterior and posterior corona radiata and the cingulum. In addition, both PBD and NPBD shared widespread MD increases across the posterior corona radiata, cingulum, and sagittal stratum. These findings suggest that widespread WM microstructural alterations might be a common neuroanatomical characteristic of bipolar disorder, regardless of being psychotic or non-psychotic. Particularly, PBD might involve extensive inter-and intra-hemispheric WM connectivity disruptions.

## 1. Introduction

Bipolar disorder (BD) is a disabling neuropsychiatric illness characterized by recurrent manic and depressive episodes [1]. Since Kraepelin’s concept of manic-depressive insanity, BD had historically been considered a mood disorder along with major depressive disorder [2]. However, BD is highly heterogeneous in its clinical manifestations, often presenting schizophrenia-like psychotic symptoms as well as mood dysregulation [3]. Moreover, large-scale genetic studies have provided strong evidence for common genetic causes between BD and schizophrenia [4, 5], while neuroimaging studies have shown shared structural abnormalities between the two disorders [6]. Considering this evidence, the concept of a psychosis continuum linking BD and schizophrenia has been recently highlighted [7, 8].

A significant proportion of patients (approximately 70% of patients with BD type I) have experienced frank psychotic symptoms, such as hallucination and delusion, during their illness [9]. Particularly, compared to non-psychotic BD (NPBD), psychotic BD (PBD) is characterized by earlier onset, poorer treatment response, more relapse, and lower cognitive and social function, also common in patients with schizophrenia [10]. Thus, from the point of view of a psychosis continuum, PBD has been expected to be closer to psychotic disorders such as schizophrenia, whereas NPBD might be closer to affective disorders [11]. However, the degree of shared biological underpinnings between PBD and schizophrenia and the differences in neuroanatomical characteristics between PBD and NPBD remain unclear. Accordingly, exploring the neural substrates of the psychotic features in BD could improve our understanding of the neurobiological basis of the psychosis continuum clinical concept.

Diffusion tensor imaging (DTI) studies have revealed extensive white matter (WM) abnormalities not only in schizophrenia [12] but also in BD [13, 14]. Recent comparative studies evidenced an overlap of affected WM areas in BD and schizophrenia [15, 16]. However, few studies have investigated the neural substrates associated with psychotic features in BD, and little is known about the patterns of WM abnormalities in PBD and NPBD. Therefore, this study aimed to concurrently investigate WM alterations in PBD and NPBD using tract-based spatial statistical (TBSS) analyses and explore WM microstructural differences between the two BD subtypes. Particularly, we hypothesized that PBD might involve extensive WM abnormalities, possibly comparable to those of schizophrenia. This is in line with evidence of more extensive gray matter volume deficits in PBD than in NPBD [17, 18].

## 2. Materials and methods

### 2.1. Subjects

All subjects were recruited from outpatient clinics of the National Center for Mental Health, Seoul, Korea, and a psychologist interviewed them using the Mini-International Neuropsychiatric Interview (MINI) [19]. The diagnosis of BD was assessed through the Diagnostic and Statistical Manual of Mental Disorders, Fourth Edition (DSM-IV) criteria and confirmed by the clinical consensus of expert psychiatrists. Inclusion criteria were as follows: (1) age 20–50 years old; (2) duration of illness > 1 year; (3) no change in general clinical state and medication for ≥3 months prior to the time of assessment. We excluded patients with a concurrent axis I diagnosis according to the DSM-IV including schizophrenia, schizoaffective disorder, and alcohol use disorder, current or past neurological disease, any contraindication to MRI scan, or a physical condition that would render an MRI scan difficult to administer or interpret. According to the K module in the MINI [19], patients with lifetime experience of psychotic symptoms were assigned into the PBD group, while the others were assigned to the NPBD group. The healthy control (HC) group consisted of volunteers from the local community in the same age range and without history of psychiatric disorders. The same exclusion criteria for the patients was also applied to the HC group with regards to medical, neurological, and physical conditions. We assessed the patients’ overall psychopathology and functioning level using the 18-item Brief Psychiatric Rating Scale (BPRS-18) [20], Clinician-Rated Dimensions of Psychosis Symptom Severity (CRDPSS) [21], and WHO Disability Assessment Schedule 2.0 (WHODAS 2.0) [22].

We first selected 31 PBD, 31 NPBD, and 65 HC, who were recruited in order, except for three patients with BD who were excluded due to poor information or imaging. According to the MINI, among the patients, two PBD and eight NPBD were classified as BD type II, and the remaining were classified as BD type I. In addition, none of the patients had taken any substance that could induce psychosis. For subject homogeneity, we included only 52 patients with BD type I. Finally, 29 PBD, 23 NPBD, and 65 HC were included in this study.

This study was approved by the Institutional Review Board of the National Center for Mental Health (IRB approval number: 116271-2017-26), and written informed consent obtained from all subjects.

### 2.2. MRI data acquisition

MRI data were acquired using a 3-Tesla MRI scanner (Ingenia CX; Philips, Erlangen) equipped with a 32-channel head coil at National Center for Mental Health. A diffusion-weighted image was acquired using single-shot echo-planar imaging sequence with the following parameters: acquisition matrix = 128 × 128, voxel size = 1.75 × 1.75 × 2 mm^3^, axial slices = 72, FOV = 224 × 224 mm^2^, TE = 90 ms, TR = 9000 ms, flip angle = 90°, slice gap = 0 mm, b-value = 0 and 1000 s/mm^2^, and diffusion sensitive gradient direction = 64. The baseline image without weighting was used [0, 0, 0].

### 2.3. DTI data processing

DTI processing was performed using the FMRIB Software Library (FSL) (https://fsl.fmrib.ox.ac.uk/fsl/fslwiki), ver. 6.0. First, motion artifacts and eddy current distortions through affine registration were corrected by considering the B0 volume as a reference using FSL’s Diffusion Toolbox. Then, the diffusion-weighted images were skull stripped using the Brain Extraction Tool within the FSL. Fractional anisotropy (FA) and mean diffusivity (MD) images were obtained from the tensors’ eigenvalues using the DTIFIT program in the FSL. Next, voxel-wise statistical analysis of FA and MD images was performed using the TBSS pipeline [23]. FA is the most widely used scalar in DTI, a measure indicating the overall directionality of water diffusion, higher in organized WM tracts and lower in disorganized fibers [24]. Axon damage or demyelination results in water motion being more isotropic, which may manifest in low FA values. MD describes the rotationally invariant magnitude of water diffusion within brain tissue, independent of tissue directionality [25]. MD is a non-specific, albeit sensitive, measure that can be affected by any disease process affecting the barriers restricting water movement and is usually higher in damaged tissue with edema or necrosis, for instance. FA images were aligned into the standard space (FMRIB58_FA, 1 × 1 × 1 mm MNI 152 space) using the nonlinear registration tool (FNIRT). Afterwards, a mean FA image was created and threshold by an FA value of 0.2 to exclude peripheral tracts and GM regions. Each subject’s aligned FA images were then projected onto the skeleton by filling it with the highest FA values from the nearest relevant center of the fiber tracts. The same transformation and warped-field were applied to MD images.

### 2.4. Statistical analyses

TBSS analyses were performed using the FSL toolbox (randomize). Pairwise comparisons of FA and MD values were performed at the voxel-level for PBD vs. NPBD, PBD vs. HC, NPBD vs. HC, and BD (PBD + NPBD) vs. HC using a generalized linear model. We adjusted for age, sex, education, and handedness as covariates to compare DTI parameters. To adjust the voxel-wise multiple comparisons, we adopted the family-wise error (FWE) approach. Significance thresholding for the TBSS analysis was determined using 10,000 permutations and threshold-free cluster-enhancement with the 2D parameter settings [26]. Thereafter, to adjust the number of comparisons of two DTI parameters (FA and MD) between four group pairs (PBD vs. NPBD, PBD vs. HC, NPBD vs. HC, and BD vs. HC), we applied the Bonferroni correction to the FWE-corrected P-value. Thus, the threshold of statistical significance for the TBSS analysis was conservatively set at an FWE-corrected P-value < 0.0062 (0.05 / 8) with a cluster size > 100 mm^3^.

In addition to group comparisons, we conducted exploratory analyses to investigate the association of DTI parameters with severity of psychopathology and functioning level, represented by the BPRS-18 and WHODAS 2.0 total scores, respectively, in each PBD, NPBD, and BD group. For these analyses, we adjusted for sex, age, education, handedness, and chlorpromazine-equivalent dose of antipsychotic drug, applying an FWE-corrected P-value < 0.05.

For comparisons of demographic and clinical data, a P-value < 0.05 was considered statistically significant.

## 3. Results

### 3.1. Demographic and clinical characteristics

Table 1 summarizes the demographic and clinical characteristics of the PBD, NPBD, and HC groups. There were no significant differences in age (F = 0.34, P = 0.711), sex (χ^2^ = 4.36, P = 0.113), level of education (F = 2.55, P = 0.083), and handedness (χ^2^ = 0.25, P = 0.884) among the three groups, as well as in the duration of illness (t = 1.05, P = 0.298) and chlorpromazine-equivalent dose of antipsychotic drugs (t = 1.70, P = 0.096) between the PBD and NPBD groups. According to the BPRS-18 and WHODAS 2.0 scores, PBD and NPBD were clinically stable without overall vivid psychotic symptoms and their social functioning was also well preserved at the time of assessments. In particular, the scores of CRDPSS domains showed that most PBD and NPBD had been experiencing less than a mild level of manic or depressive symptoms. There were also no significant differences in severity of psychopathology and functioning level between the PBD and NPBD groups. In addition, there were no significant differences in age (t = 0.83, P = 0.409), sex (χ^2^ = 3.94, P = 0.063), and handedness (χ^2^ = 0.21, P = 0.771) between the BD group and HC. However, the level of education was significantly lower in the BD group than in HC (t = -2.18, P = 0.032).

**Table 1:** Demographic and clinical characteristics.

Variables^a^	Psychotic bipolar disorder	Non-psychotic bipolar disorder	Healthy control	Statistics^b^
	(N = 29)	(N = 23)	(N = 65)	
Age, y	35.90 ± 7.33	35.78 ± 8.93	34.52 ± 8.93	F = 0.34, P = 0.711
Sex (male / female), n	19 / 10	13 / 10	28 / 37	χ^2^ = 4.36, P = 0.113
Education, y	14.14 ± 1.77	13.61 ± 1.88	14.78 ± 2.55	F = 2.55, P = 0.083
Handedness (right / left), n	26 / 3	21 / 2	57 / 8	χ^2^ = 0.25, P = 0.884
Duration of illness, y	13.71 ± 8.15	11.65 ± 7.31	-	t = 1.05, P = 0.298
Antipsychotics, n	29	22	-	χ^2^ = 1.29, P = 0.442
Chlorpromazine-equivalent dose, mg	516.25 ± 315.08	370.23 ± 301.52	-	t = 1.70, P = 0.096
Mood stabilizers, n	20	20	-	χ^2^ = 2.34, P = 0.188
valproate / lithium / lamotrigine, n	14 / 7 / 3	14 / 7 / 1	-	-
Antidepressants, n	1	4	-	χ^2^ = 2.87, P = 0.157
BPRS-18 total score	27.24 ± 7.49	27.74 ± 8.17	-	t = -0.23, P = 0.820
BPRS-18 subscale scores^c^				
Affect	7.76 ± 3.25	8.09 ± 3.94	-	t = -0.33, P = 0.743
Positive symptoms	5.72 ± 3.07	5.48 ± 2.04	-	t = 0.33, P = 0.743
Negative symptoms	5.21 ± 2.16	4.78 ± 2.43	-	t = 0.66, P = 0.509
Resistance	3.66 ± 1.26	4.43 ± 1.93	-	t = -1.68, P = 0.102
Activation	3.90 ± 1.40	4.09 ± 1.50	-	t = -0.47, P = 0.639
CRDPSS total score	3.66 ± 3.24	4.35 ± 3.76	-	t = -0.71, P = 0.479
CRDPSS domain scores				
Hallucination	0 (0–0)	0 (0–0)	-	U = 324.50, P = 0.682
Delusion	0 (0–0)	0 (0–1)	-	U = 292.00, P = 0.313
Disorganized speech	0 (0–0)	0 (0–1)	-	U = 306.00, P = 0.477
Abnormal psychomotor behavior	0 (0–0)	0 (0–0)	-	U = 316.00, P = 0.425
Negative symptom	1 (0–2)	0 (0–1)	-	U = 305.00, P = 0.566
Impaired cognition	1 (0–1)	1 (0–2)	-	U = 261.50, P = 0.155
Depression	1 (0–2)	1 (0–2)	-	U = 306.50, P = 0.593
Mania	0 (0–1)	0 (0–1)	-	U = 325.00, P = 0.850
WHODAS 2.0 total score	10.01 ± 7.67	11.61 ± 7.45	-	t = -0.75, P = 0.454
WHODAS 2.0 domain scores				
Cognition	10.78 ± 11.44	12.86 ± 10.65	-	t = -0.68, P = 0.500
Mobility	1.21 ± 3.18	3.04 ± 5.98	-	t = -1.42, P = 0.161
Self-care	2.59 ± 3.92	2.72 ± 4.55	-	t = -0.11, P = 0.912
Getting along	21.38 ± 17.57	21.96 ± 16.22	-	t = -0.12, P = 0.904
Life activities	6.47 ± 7.33	9.65 ± 8.53	-	t = -1.45, P = 0.154
Participation	17.67 ± 12.81	19.43 ± 10.23	-	t = -0.54, P = 0.595

^a^ Data are shown as mean ± standard deviation, number, or median (interquartile range).

^b^ ANOVA, Fisher exact test, independent t test, or Mann-Whitney test.

^c^ Factor structures proposed by Shafer (2005) [27].

Abbreviations: BPRS-18, 18-item Brief Psychiatric Rating Scale; CRDPSS, Clinician-Rated Dimensions of Psychosis Symptom Severity; WHODAS 2.0, WHO Disability Assessment Schedule 2.0.

### 3.2. FA and MD comparison between groups

**Fig 1:**
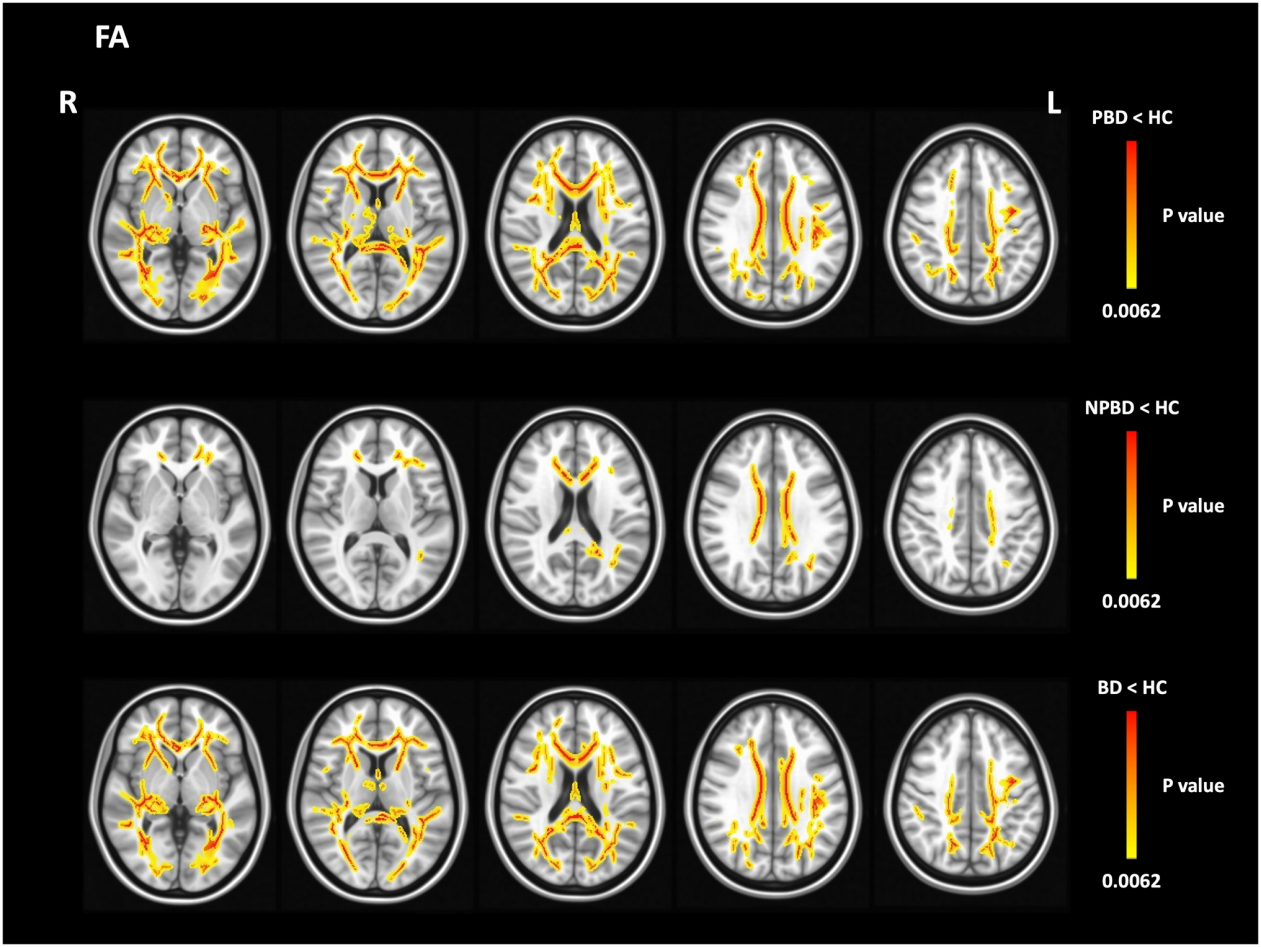
Tract-based spatial statistics analyses identified white matter areas affected by a significant decrease in fractional anisotropy (FA) in patients with psychotic bipolar disorder (PBD) (N = 29), non-psychotic bipolar disorder (NPBD) (N = 23), and bipolar disorder (BD) (N = 52) in comparisons with healthy controls (HCs) (N = 65). Family-wise error corrected P < 0.0062.

Comparisons of FA between PBD and NPBD revealed no significant differences. However, compared with HC, PBD showed widespread FA decreases in the body and splenium of the corpus callosum as well as in the bilateral internal capsule and fornix (Fig 1 and Table 2). NPBD showed local FA decreases in a part of the corpus callosum body and limited regions within the left cerebral hemisphere, including the anterior and posterior corona radiata and cingulum.

**Table 2:** Comparisons of fractional anisotropy among PBD (N = 29), NPBD (N = 23), BD (PBD + NPBD) (N = 52), and HC (N = 65) groups^a^.

Anatomical region	Side	T max	Peak coordinates (MNI)			Cluster size
			x	y	z	(mm^3^)
PBD vs. HC						
Body of corpus callosum		6.873	-10	17	22	30,764
Retrolenticular part of internal capsule	L	4.707	-29	-24	5	1787
Fornix (column and body)		4.300	0	6	6	625
Splenium of corpus callosum		3.960	-2	-34	14	355
Anterior limb of internal capsule	R	2.728	12	5	4	120
NPBD vs. HC						
Body of corpus callosum		5.447	-10	19	21	4291
Posterior corona radiata	L	5.532	-17	-53	31	579
Anterior corona radiata	L	4.047	-26	33	6	456
Cingulum	L	5.085	-7	-10	35	346
BD vs. HC						
Body of corpus callosum		6.807	-9	17	22	25,656
Posterior thalamic radiation	R	4.880	32	-64	1	3908
Retrolenticular part of internal capsule	L	4.441	-29	-22	3	1451
Posterior corona radiata	R	3.598	37	-57	23	423
Splenium of corpus callosum		3.759	-7	-39	15	365
Sagittal stratum	L	3.813	-49	-24	-17	286
Superior longitudinal fasciculus	R	3.030	42	-43	9	165
Posterior corona radiata	L	3.618	-26	-33	25	115

^a^ Family-wise error corrected P < 0.0062

Note that all MNI coordinates of maximum t values are selected in the significant region.

Abbreviations: PBD, Psychotic Bipolar Disorder; NPBD, Non-Psychotic Bipolar Disorder; BD, Bipolar Disorder; HC, Healthy Control; MNI, Montreal Neurological Institute; L, Left; R, Right.

Compared with HC, all patients with BD also showed widespread FA decreases in the right posterior thalamic radiation, right posterior corona radiata, and left sagittal stratum as well as the corpus callosum, left retrolenticular part of the internal capsule, and the left posterior corona radiata, which were also affected in PBD and NPBD (Fig 1 and Table 2).

**Fig 2:**
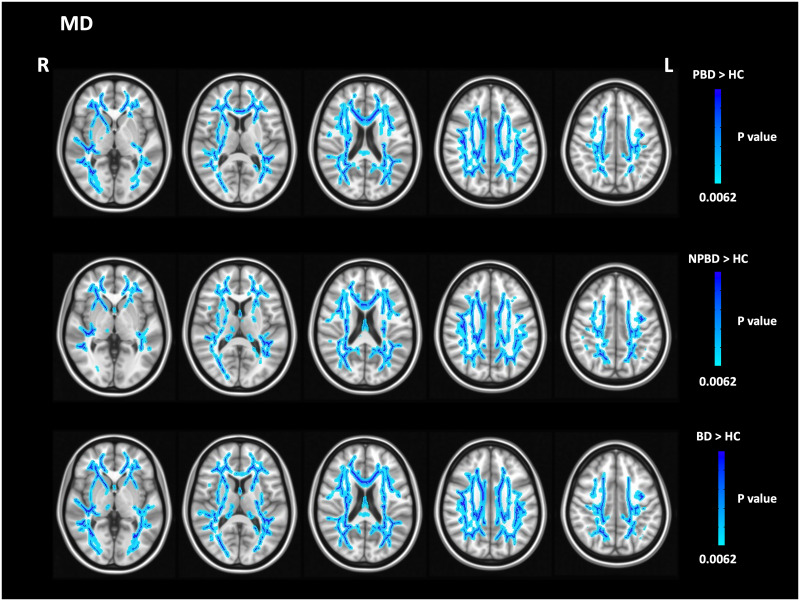
Tract-based spatial statistics analyses identified white matter areas affected by a significant increase in mean diffusivity (MD) in patients with psychotic bipolar disorder (PBD) (N = 29), non-psychotic bipolar disorder (NPBD) (N = 23), and bipolar disorder (BD) (N = 52) in comparisons with healthy controls (HCs) (N = 65). Family-wise error corrected P < 0.0062.

Comparisons of MD between PBD and NPBD revealed no significant differences. However, compared with HC, PBD and NPBD showed a widespread MD increase mainly in the right superior corona radiate (Fig 2 and Table 3). In addition, PBD showed MD increases across the left posterior corona radiata, left posterior thalamic radiation, left cingulum, left crus of fornix, and bilateral sagittal stratum. NPBD also showed MD increases across the right retrolenticular part of internal capsule, left superior longitudinal fasciculus, bilateral cingulum, and left sagittal stratum.

**Table 3:** Comparisons of mean diffusivity among PBD (N = 29), NPBD (N = 23), BD (PBD + NPBD) (N = 52), and HC (N = 65) groups^a^.

Anatomical region	Side	T max	Peak coordinates (MNI)			Cluster size
			x	y	Z	(mm^3^)
PBD vs. HC						
Superior corona radiata	R	6.704	28	7	27	35,366
Posterior corona radiata	L	5.010	-17	-51	32	604
Posterior thalamic radiation	L	3.911	-36	-52	11	429
Cingulum	L	5.045	-8	-5	33	416
Fornix (crus)	L	5.079	-34	-9	-16	366
Sagittal stratum	R	4.019	41	-38	-10	198
Sagittal stratum	L	3.373	-40	-42	-7	114
Anterior limb of internal capsule	L	3.649	-22	-4	16	107
Retrolenticular part of internal capsule	L	4.019	-29	-23	-1	103
NPBD vs. HC						
Superior corona radiata	R	6.222	28	6	29	27,510
Retrolenticular part of internal capsule	R	5.217	41	-29	3	912
Superior longitudinal fasciculus	L	5.466	-44	-1	27	748
Cingulum	L	4.705	-11	-48	23	377
Sagittal stratum	L	3.723	-40	-29	-16	270
External capsule	R	4.480	32	-2	12	169
Cingulum	R	4.436	9	16	28	141
Fornix (crus)	R	5.250	11	-47	25	107
BD vs. HC						
Superior corona radiata	R	7.465	28	7	28	40,579
Fornix (column and body)		5.579	3	-11	15	783
Cingulum	L	4.893	-8	-5	33	489
Posterior thalamic radiation	R	4.394	36	-56	-6	339
Retrolenticular part of internal capsule	L	3.189	-29	-23	-1	186
External capsule	R	4.602	35	-5	-1	143
External capsule	L	3.453	-31	-16	12	129
Superior corona radiata	L	3.216	-27	-7	20	102

^a^ Family-wise error corrected P < 0.0062

Note that all MNI coordinates of maximum t values are selected in the significant region.

Abbreviations: PBD, Psychotic Bipolar Disorder; NPBD, Non-Psychotic Bipolar Disorder; BD, Bipolar Disorder; HC, Healthy Control; MNI, Montreal Neurological Institute; L, Left; R, Right.

Compared with HC, all patients with BD also showed widespread MD increases in the column and body of the fornix and right posterior thalamic radiation as well as in the right superior corona radiata and left cingulum, which were also affected in PBD and NPBD (Fig 2 and Table 3).

In addition, we found no significant association between DTI parameters and continuous clinical variables, including the BPRS-18 and WHODAS 2.0 total scores, in all patients with BD as well as in PBD and NPBD.

## 4. Discussion

This study investigated WM microstructural alterations in PBD and NPBD using TBSS analyses. We observed no significant difference in diffusion measures between PBD and NPBD. However, in comparison with HC, patients with PBD and NPBD exhibited widespread alterations in the WM microstructure, with possible differences between the two BD subtypes. PBD showed widespread decreases in FA, particularly in the body and splenium of the corpus callosum, as well as in the bilateral internal capsule and fornix. Conversely, NPBD showed local decreases in FA only in a part of the corpus callosum body and some WM areas within the left cerebral hemisphere. In contrast to decreased FA, both PBD and NPBD showed widespread increases in MD across the whole-brain WM skeleton.

### 4.1. Comparisons of diffusion measures between PBD and NPBD

In this study, we first compared diffusional measures between PBD and NPBD to explore WM substrates that might be unique to PBD contributing to psychotic features and poorer clinical course. However, there was no significant difference in FA and MD between PBD and NPBD. Similar to our finding, recent studies have shown minimal differences in DTI parameters between PBD and NPBD. Ji et al. found a significant FA difference between the two BD subtypes only in the left uncinate fasciculus [28]. Furthermore, Brown et al. could not find any difference in diffusion measures between PBD and NPBD [29]. The sample size of these studies, and our study, might have been too small to detect subtle differences between the two BD subtypes. In addition, these studies revealed significant diffusional alterations in patients with PBD and NPBD compared with HC [28, 29]. These microstructural alterations have mainly been interpreted as the WM pathology associated with affective disturbance in BD. However, we supposed that the distribution pattern of WM abnormalities might be different between PBD and NPBD, which might be a neuroanatomical characteristic underlying the different clinical manifestations between the two BD subtypes.

### 4.2. Widespread compromise of WM integrity and connectivity in PBD

In the present study, compared with HC, PBD showed a pronounced FA decrease in the corpus callosum, with widespread FA decreases across the internal capsule and fornix. PBD also showed widespread WM areas affected by MD increase across the right superior corona radiata, left posterior thalamic radiation, and left cingulum. Considering that either a decrease in FA or an increase in MD reflects disruptions to water diffusion coherence due to axonal degradation, demyelination, or neurodegeneration [25], these findings suggest that PBD might involve widespread alterations in the WM microstructure across cortico-cortical, cortico-limbic, thalamocortical, and callosal connections. These patterns of widespread WM abnormalities are also largely in agreement with the results of previously published large DTI analyses and meta-analyses of schizophrenia, which provided evidence of extensive disruptions of WM integrity and connectivity across frontotemporal, frontosubcortical, and callosal networks in patients of schizophrenia [30–32]. In particular, given that the corpus callosum contains axon fibers connecting the bilateral frontal cortices and that WM integrity is related to cognitive performance in various domains including sustained attention, processing speed, and problem solving abilities [33], extensive disruptions of WM connectivity in the corpus callosum might be the underlying mechanism of the more severe cognitive decline in PBD compared to that in NPBD [10]. Therefore, this study’s findings suggest that extensive WM integrity and connectivity disruptions might be the neural substrates underlying the clinical manifestations of PBD, such as concurrent psychotic features, cognitive impairment, and poor clinical outcome, overlapping with those of patients with schizophrenia, largely in line with our prior hypothesis that PBD might be close to schizophrenia on a psychosis continuum.

### 4.3. Local disruptions of WM connectivity along with widespread microstructural alterations in NPBD

We observed that, compared with HC, NPBD showed local decreases in FA in a part of the corpus callosum and some WM areas within the left cerebral hemisphere, including the anterior and posterior corona radiata and cingulum. However, the increase in MD was more widely distributed across both cerebral hemispheres, including the right superior corona radiata, right retrolenticular part of internal capsule, left superior longitudinal fasciculus, and left cingulum, which partially overlapped with the affected WM areas in PBD. FA measures tract directional coherence, and a decrease in FA indicates impaired WM connectivity [34]. However, MD is directionally averaged and consequently less influenced by directional coherence [35]. Thus, our findings suggest that NPBD might involve widespread WM microstructural alterations, but WM connectivity might be compromised only in a part of the corpus callosum body and limited WM areas within the left cerebral hemisphere. In addition, major depression has been reportedly associated with microstructural alterations in the affected WM areas in NPBD in this study, particularly, the corpus callosum and corona radiata [36, 37]. There is also evidence of brain asymmetry, such as left hemispheric hypo-activation or right hemispheric hyper-activation, in patients with depression or mania [38–40]. In this respect, impairments of inter- and intra-hemispheric connectivity within the left cerebral hemisphere could be interpreted as a possible neural mechanism underlying the affective symptoms in patients with BD. Further studies to investigate hemispheric differences in the pathophysiology of BD are warranted.

### 4.4. Widespread compromise of WM integrity in BD

In this study, comparisons of HC with total BD patients, including both PBD and NPBD, showed widespread alterations of DTI parameters in major WM tracts. This is largely consistent with the results of previous DTI studies reporting extensive impairment of WM integrity across the cortico-cortical, cortico-subcortical, and callosal networks in BD [13, 14]. Thus, these findings implicate BD as a brain network disorder involving widespread compromise of WM integrity. However, it has not been elucidated whether the disruptions of WM integrity are common to both PBD and NPBD or whether they are more pronounced in PBD. Given that most patients with BD experience psychotic features in their lifetime [9], previous BD studies might have included a high proportion of patients with PBD. Thus, we supposed that the extensive WM integrity impairment reported in the previous studies might have been associated with psychotic features in patients with BD as well as their affective disturbance. In this study, comparisons between patients with PBD and HC revealed FA decrease, widely distributed across both cerebral hemispheres and a large portion of the corpus callosum. In contrast, in NPBD, this decrease was limited only to local regions within the left cerebral hemisphere and corpus callosum body. Considering that a decrease in FA reflects decreased axonal connectivity [25, 34], these findings suggest that PBD might involve extensive disruptions of inter-and intra-hemispheric WM connectivity, contributing to its psychotic features and poor clinical outcomes. However, in contrast to FA, PBD and NPBD shared increases in MD across both cerebral hemispheres, reflecting widespread alterations in axonal water diffusion in both BD subtypes. Therefore, the patterns of WM microstructural alterations in PBD and NPBD in this study should be interpreted carefully. In addition, future studies need to investigate the neurobiological substrates underlying BD by dividing the patients according to the presence or absence of psychotic features.

### 4.5. Limitations

This study has some methodological limitations. First, study participants had a wide range of illness duration and had received various types and doses of antipsychotics, mood stabilizers, or antidepressants. Considering the evidence on the effects of aging, disease severity, and medications on WM integrity [41], we cannot exclude the possibility that subject heterogeneity might have influenced the results. Second, the sample size for the PBD and NPBD groups was small and was not matched; therefore, the statistical power may not be sufficient to detect differences in the direct comparison of the two groups. Third, we could not find any significant association between illness severity and diffusion measures, probably because most patients were clinically stable with less than a mild level of mood symptoms and functional impairment.

### 4.6 Conclusion

In conclusion, widespread alterations in the WM microstructure might be a common neuroanatomical characteristic of BD, regardless of PBD and NPBD. In particular, PBD might involve extensive disruptions of inter- and intra-hemispheric WM connectivity, suggesting that psychotic features in patients with BD might be attributed to severe impairment in brain networks. Furthermore, these findings could improve our understanding of the pathophysiological basis underlying the clinical and neurobiological continuum of psychosis. Future studies to investigate the underlying neural mechanism of psychotic features in patients with BD are warranted.

## References

[1] Phillips ML, Kupfer DJ. Bipolar disorder diagnosis: challenges and future directions. Lancet. 2013; 381:1663–71. doi: 10.1016/S0140-6736(13)60989-7 23663952 PMC5858935

[2] Rybakowski JK. 120th Anniversary of the Kraepelinian Dichotomy of Psychiatric Disorders. Curr Psychiatry Rep. 2019; 21:65. doi: 10.1007/s11920-019-1048-6 31264045 PMC6603189

[3] Tamminga CA, Ivleva EI, Keshavan MS, Pearlson GD, Clementz BA, Witte B, et al. Clinical phenotypes of psychosis in the Bipolar-Schizophrenia Network on Intermediate Phenotypes (B-SNIP). Am J Psychiatry. 2013; 170:1263–74. doi: 10.1176/appi.ajp.2013.12101339 23846857 PMC12866513

[4] Bipolar D, Schizophrenia Working Group of the Psychiatric Genomics Consortium. Electronic address drve, Bipolar D, Schizophrenia Working Group of the Psychiatric Genomics C. Genomic Dissection of Bipolar Disorder and Schizophrenia, Including 28 Subphenotypes. Cell. 2018; 173:1705–15.e16. doi: 10.1016/j.cell.2018.05.046 29906448 PMC6432650

[5] International Schizophrenia C, Purcell SM, Wray NR, Stone JL, Visscher PM, O’Donovan MC, et al. Common polygenic variation contributes to risk of schizophrenia and bipolar disorder. Nature. 2009; 460:748–52. doi: 10.1038/nature08185 19571811 PMC3912837

[6] Ellison-Wright I, Bullmore E. Anatomy of bipolar disorder and schizophrenia: a meta-analysis. Schizophr Res. 2010; 117:1–12. doi: 10.1016/j.schres.2009.12.022 20071149

[7] Craddock N, Owen MJ. The beginning of the end for the Kraepelinian dichotomy. Br J Psychiatry. 2005; 186:364–6. doi: 10.1192/bjp.186.5.364 15863738

[8] Craddock N, Owen MJ. The Kraepelinian dichotomy—going, going… but still not gone. Br J Psychiatry. 2010; 196:92–5. doi: 10.1192/bjp.bp.109.073429 20118450 PMC2815936

[9] Dunayevich E, Keck PE Jr. Prevalence and description of psychotic features in bipolar mania. Curr Psychiatry Rep. 2000; 2:286–90. doi: 10.1007/s11920-000-0069-4 11122970

[10] van Bergen AH, Verkooijen S, Vreeker A, Abramovic L, Hillegers MH, Spijker AT, et al. The characteristics of psychotic features in bipolar disorder. Psychol Med. 2019; 49:2036–48. doi: 10.1017/S0033291718002854 30303059

[11] Karlsgodt KH. White Matter Microstructure across the Psychosis Spectrum. Trends Neurosci. 2020; 43:406–16. doi: 10.1016/j.tins.2020.03.014 32349908 PMC7676818

[12] Kelly S, Jahanshad N, Zalesky A, Kochunov P, Agartz I, Alloza C, et al. Widespread white matter microstructural differences in schizophrenia across 4322 individuals: results from the ENIGMA Schizophrenia DTI Working Group. Mol Psychiatry. 2018; 23:1261–9. doi: 10.1038/mp.2017.170 29038599 PMC5984078

[13] Barysheva M, Jahanshad N, Foland-Ross L, Altshuler LL, Thompson PM. White matter microstructural abnormalities in bipolar disorder: A whole brain diffusion tensor imaging study. Neuroimage Clin. 2013; 2:558–68. doi: 10.1016/j.nicl.2013.03.016 24179807 PMC3777761

[14] Favre P, Pauling M, Stout J, Hozer F, Sarrazin S, Abe C, et al. Widespread white matter microstructural abnormalities in bipolar disorder: evidence from mega- and meta-analyses across 3033 individuals. Neuropsychopharmacology. 2019; 44:2285–93. doi: 10.1038/s41386-019-0485-6 31434102 PMC6898371

[15] Dong D, Wang Y, Chang X, Jiang Y, Klugah-Brown B, Luo C, et al. Shared abnormality of white matter integrity in schizophrenia and bipolar disorder: A comparative voxel-based meta-analysis. Schizophr Res. 2017; 185:41–50. doi: 10.1016/j.schres.2017.01.005 28082140

[16] Squarcina L, Bellani M, Rossetti MG, Perlini C, Delvecchio G, Dusi N, et al. Similar white matter changes in schizophrenia and bipolar disorder: A tract-based spatial statistics study. PLoS One. 2017; 12:e0178089. doi: 10.1371/journal.pone.0178089 28658249 PMC5489157

[17] Altamura AC, Maggioni E, Dhanoa T, Ciappolino V, Paoli RA, Cremaschi L, et al. The impact of psychosis on brain anatomy in bipolar disorder: A structural MRI study. J Affect Disord. 2018; 233:100–9. doi: 10.1016/j.jad.2017.11.092 29223329

[18] Ekman CJ, Petrovic P, Johansson AG, Sellgren C, Ingvar M, Landen M. A History of Psychosis in Bipolar Disorder is Associated With Gray Matter Volume Reduction. Schizophr Bull. 2017; 43:99–107. doi: 10.1093/schbul/sbw080 27289116 PMC5216851

[19] Sheehan DV, Lecrubier Y, Sheehan KH, Amorim P, Janavs J, Weiller E, et al. The Mini-International Neuropsychiatric Interview (M.I.N.I.): the development and validation of a structured diagnostic psychiatric interview for DSM-IV and ICD-10. J Clin Psychiatry. 1998; 59 Suppl 20:22–33;quiz 4–57. 9881538

[20] Overall JE, Gorham DR. The brief psychiatric rating scale. Psychol Rep. 1962; 10:799–812.

[21] Barch DM, Bustillo J, Gaebel W, Gur R, Heckers S, Malaspina D, et al. Logic and justification for dimensional assessment of symptoms and related clinical phenomena in psychosis: relevance to DSM-5. Schizophr Res. 2013; 150:15–20. doi: 10.1016/j.schres.2013.04.027 23706415

[22] Gold LH. DSM-5 and the assessment of functioning: the World Health Organization Disability Assessment Schedule 2.0 (WHODAS 2.0). J Am Acad Psychiatry Law. 2014; 42:173–81. 24986344

[23] Smith SM, Jenkinson M, Johansen-Berg H, Rueckert D, Nichols TE, Mackay CE, et al. Tract-based spatial statistics: voxelwise analysis of multi-subject diffusion data. Neuroimage. 2006; 31:1487–505. doi: 10.1016/j.neuroimage.2006.02.024 16624579

[24] Tae WS, Ham BJ, Pyun SB, Kang SH, Kim BJ. Current Clinical Applications of Diffusion-Tensor Imaging in Neurological Disorders. J Clin Neurol. 2018; 14:129–40. doi: 10.3988/jcn.2018.14.2.129 29504292 PMC5897194

[25] Clark KA, Nuechterlein KH, Asarnow RF, Hamilton LS, Phillips OR, Hageman NS, et al. Mean diffusivity and fractional anisotropy as indicators of disease and genetic liability to schizophrenia. J Psychiatr Res. 2011; 45:980–8. 21306734 10.1016/j.jpsychires.2011.01.006 PMC3109158

[26] Smith SM, Johansen-Berg H, Jenkinson M, Rueckert D, Nichols TE, Miller KL, et al. Acquisition and voxelwise analysis of multi-subject diffusion data with tract-based spatial statistics. Nat Protoc. 2007; 2:499–503. doi: 10.1038/nprot.2007.45 17406613

[27] Shafer A. Meta-analysis of the brief psychiatric rating scale factor structure. Psychological assessment. 2005; 17:324. doi: 10.1037/1040-3590.17.3.324 16262458

[28] Ji A, Godwin D, Rutlin J, Kandala S, Shimony JS, Mamah D. Tract-based analysis of white matter integrity in psychotic and nonpsychotic bipolar disorder. J Affect Disord. 2017; 209:124–34. doi: 10.1016/j.jad.2016.11.038 27914246

[29] Brown JA, Jackson BS, Burton CR, Hoy JE, Sweeney JA, Pearlson GD, et al. Reduced white matter microstructure in bipolar disorder with and without psychosis. Bipolar Disorders. 2021; 23:801–9. doi: 10.1111/bdi.13055 33550654 PMC8514149

[30] Bora E, Fornito A, Radua J, Walterfang M, Seal M, Wood SJ, et al. Neuroanatomical abnormalities in schizophrenia: a multimodal voxelwise meta-analysis and meta-regression analysis. Schizophr Res. 2011; 127:46–57. doi: 10.1016/j.schres.2010.12.020 21300524

[31] Wheeler AL, Voineskos AN. A review of structural neuroimaging in schizophrenia: from connectivity to connectomics. Front Hum Neurosci. 2014; 8:653. doi: 10.3389/fnhum.2014.00653 25202257 PMC4142355

[32] Patel S, Mahon K, Wellington R, Zhang J, Chaplin W, Szeszko PR. A meta-analysis of diffusion tensor imaging studies of the corpus callosum in schizophrenia. Schizophr Res. 2011; 129:149–55. doi: 10.1016/j.schres.2011.03.014 21530178

[33] Duncan J, Owen AM. Common regions of the human frontal lobe recruited by diverse cognitive demands. Trends Neurosci. 2000; 23:475–83. doi: 10.1016/s0166-2236(00)01633-7 11006464

[34] Beaulieu C. The basis of anisotropic water diffusion in the nervous system—a technical review. NMR Biomed. 2002; 15:435–55. doi: 10.1002/nbm.782 12489094

[35] Alexander AL, Hurley SA, Samsonov AA, Adluru N, Hosseinbor AP, Mossahebi P, et al. Characterization of cerebral white matter properties using quantitative magnetic resonance imaging stains. Brain Connect. 2011; 1:423–46. doi: 10.1089/brain.2011.0071 22432902 PMC3360545

[36] Chen G, Hu X, Li L, Huang X, Lui S, Kuang W, et al. Disorganization of white matter architecture in major depressive disorder: a meta-analysis of diffusion tensor imaging with tract-based spatial statistics. Sci Rep. 2016; 6:21825. doi: 10.1038/srep21825 26906716 PMC4764827

[37] van Velzen LS, Kelly S, Isaev D, Aleman A, Aftanas LI, Bauer J, et al. White matter disturbances in major depressive disorder: a coordinated analysis across 20 international cohorts in the ENIGMA MDD working group. Mol Psychiatry. 2020; 25:1511–25. doi: 10.1038/s41380-019-0477-2 31471575 PMC7055351

[38] Bruder GE, Stewart JW, Hellerstein D, Alvarenga JE, Alschuler D, McGrath PJ. Abnormal functional brain asymmetry in depression: evidence of biologic commonality between major depression and dysthymia. Psychiatry Res. 2012; 196:250–4. doi: 10.1016/j.psychres.2011.11.019 22397909 PMC3361602

[39] Bruder GE, Stewart JW, McGrath PJ. Right brain, left brain in depressive disorders: Clinical and theoretical implications of behavioral, electrophysiological and neuroimaging findings. Neurosci Biobehav Rev. 2017; 78:178–91. doi: 10.1016/j.neubiorev.2017.04.021 28445740

[40] Perrin JS, Merz S, Bennett DM, Currie J, Steele DJ, Reid IC, et al. Electroconvulsive therapy reduces frontal cortical connectivity in severe depressive disorder. Proc Natl Acad Sci U S A. 2012; 109:5464–8. doi: 10.1073/pnas.1117206109 22431642 PMC3325678

[41] Cea-Canas B, de Luis R, Lubeiro A, Gomez-Pilar J, Sotelo E, Del Valle P, et al. Structural connectivity in schizophrenia and bipolar disorder: Effects of chronicity and antipsychotic treatment. Prog Neuropsychopharmacol Biol Psychiatry. 2019; 92:369–77. doi: 10.1016/j.pnpbp.2019.02.006 30790676

