# Supplementary material for: Different patterns of white matter microstructural alterations between psychotic and non-psychotic bipolar disorder
Source: PLoS One. 2022 Mar 18;17(3):e0265671. doi: 10.1371/journal.pone.0265671 (PMC8933039; doi:10.1371/journal.pone.0265671)
Supplement: Attachment — Submitted filename: Response to Reviewer.docx [file pone.0265671.s002.docx]

Reviewer #2: The authors addressed most of my concerned. However, they still present the qualitative differences between the PBD vs. NPBD as statistically significant. On p.10 of the revised manuscript, the authors clearly state that "Direct comparisons of FA and MD between PBD and NPBD revealed no significant difference". Therefore, discussing "different patterns of WM connectivity disruptions between PBD and NPBD" does not make any sense. Moreover, the Discussion section should state and discuss the fact that there were NO significant differences between psychotic and non-psychotic groups. Considering that the BD groups did not differ from each other, the authors could focus on the BD vs. HC comparison. The results are also interesting and can be discussed in the context of existing literature. These results should be reported and discussed right after the non-significant results are reported and discussed. The findings regarding the HC vs. PBD and HC vs. NPBD differences can be reported, but should not be compared as the differences are not statistically significant. The statements like "In particular, disruptions of inter- and intra- hemispheric WM connectivity, in terms of FA, might be pronounced in PBD" are not statistically supported.

**- Response: We would like to thank reviewer #2 for the careful and thoughtful suggestions. We agree with the comments and have revised our abstract and manuscript more modestly (highlighted, page 2 line 13–16, page 14 line 177‒178). First, we have added the negative findings of direct comparisons between PBD and NPBD in section 4.1 (highlighted, page 15 line 187–200). We have also discussed the results of comparisons with HC in sections 4.2 and 4.3. Finally, we have mentioned the implication of WM alterations in PBD, NPBD, and BD in section 4.4 (highlighted, page 17 line 187 – page 18 line 270). In addition, we have deleted the section on “different patterns of WM connectivity disruptions between PBD and NPBD.”**
